# Compensatory Base Changes Reveal Sexual Incompatibility among Members of the *Anopheles subpictus* Sensu Lato (Diptera: Culicidae) Species Complex in Sri Lanka

**DOI:** 10.3390/life11030211

**Published:** 2021-03-08

**Authors:** D. P. W. Jayatunga, I. N. Harischandra, N. V. Chandrasekharan, B. G. D. N. K. de Silva

**Affiliations:** 1 Center for Biotechnology, Department of Zoology, Faculty of Applied Sciences, University of Sri Jayewardenepura, Gangodawila, Nugegoda 10250, Sri Lanka; pamodajayatunga@gmail.com; 2 Genetics and Molecular Biology Unit, Faculty of Applied Sciences, University of Sri Jayewardenepura, Gangodawila, Nugegoda 10250, Sri Lanka; iresha@sci.sjp.ac.lk; 3 Department of Chemistry, Faculty of Science, University of Colombo, Colombo 00300, Sri Lanka; nchandra@chem.cmb.ac.lk

**Keywords:** *Anopheles subpictus*, compensatory base changes (CBCs), internal transcribed spacer 2 (ITS2), ITS2 secondary structures, sibling species

## Abstract

The mosquito *Anopheles (Cellia) subpictus* sensu lato (s.l.) is a major secondary vector of malaria in Sri Lanka. The sibling species composition in this species complex in Sri Lanka remains debatable. Compensatory base changes (CBCs) in the secondary structures of internal transcribed spacer 2 (ITS2) are reliable sources to predict sexual incompatibility among closely related species. The objective of the present study was to investigate the *An. subpictus* s.l. populations in Sri Lanka using the CBC analysis. Mosquito DNA was amplified and sequenced for the ITS2 region. The sequences were annotated using ITS2 Database. ITS2 secondary structures were constructed and analyzed for CBCs using various bioinformatics tools. The ITS2 regions consisted of two different lengths, 575 bp and 480 bp. The two CBCs and three hemi CBCs identified in the present study suggest that there may be at least two sexually incompatible sibling species. In conclusion, it is likely that there may be only two reproductively isolated sibling species in the *An. subpictus* species complex in Sri Lanka. However, due to high divergence of ITS2 in these species, it is reasonable to assume that they may be undergoing a speciation event to separate as a distinct species.

## 1. Introduction

Malaria is a fatal, historical disease in Sri Lanka that is caused by protozoan parasites from the genus *Plasmodium*. Having a long-documented history of malaria, the country experienced the most devastating epidemics in the period of 1934–1935 [1]. Malaria in Sri Lanka is highly unstable, such that it has historically fluctuated greatly over the years with significant seasonal differences. However, according to the Monitoring and Evaluation plan 2010–2014 of the National Malaria Control Programme of Sri Lanka, the risk of malaria outbreaks has declined dramatically [2]. As a result, Sri Lanka has been certified to be free of malaria, since 2016. However, the risk of disease recurrence on the island does not fade for being a tourist destination for travelers from malaria-endemic countries across the globe [3]. Nearly fifty cases of malaria are annually reported from various parts of the country due to imported malaria [3]; however, failure to promptly detect and treat such cases at early stages poses a serious risk of getting the disease back in the country. The presence of malaria vector mosquitoes throughout the island may be equally responsible for the potential re-emergence of the disease in all parts of the country [4].

Mosquito vectors of malaria, the Anophelines often belong to morphologically similar and reproductively isolated sympatric populations within taxons known as sibling species complexes [5]. In nomenclature, if a member sibling species in a species complex is identified and known, the suffix sensu stricto (s.s.) follows the species name. If sibling species status is unknown and a mixture of sibling species exist, the suffix sensu lato (s.l.) follows the species name. Sibling species have different habitats, biting habits and vector competence that result in different vectorial capacities [6]. Therefore, the precise identification of Anopheline mosquito species complexes plays a pivotal role in malaria elimination through vector control [7].

The main vector of malaria in Sri Lanka is *Anopheles culicifacies* Giles s.l. and *Anopheles subpictus* Grassi s.l. 1899 is a major secondary vector [8]. Eventhough *An. subpictus* is considered an important secondary vector of malaria in many parts of Sri Lanka [8], there has been no recent evidence of its *Plasmodium* transmission [9]. However, the current passive state of malaria in the country can be regarded as a warning towards future resurgences due to important secondary vectors with acquired vectorial capacity. Supportive evidence was indicated by a study conducted in Northern Jaffna district in Sri Lanka, that *An. subpictus* s.l. had a higher sporozoite rate than *An. culicifacies* during a peak transmission period [9] and also by the fact that *An. subpictus* was acting as a known malaria vector in South Asia including nearby India [10].

In India, *An. subpictus* was first found to be a species complex based on differences in larval morphology [11]. Further, based on two distinct types of eggs and cytological evidence, Reuben and Suguna [12] temporarily designated two forms of *An. subpictus* sibling species, as A and B in India. Later, four sibling species of *An. subpictus* were identified, namely A, B, C and D, based on morphological and chromosomal characters [13]. The stage-specific morphological characters to identify the siblings A–D are egg float ridge number, fourth instar larval mesothoracic seta IV, pupal seta 7-I and apical pale and pre-apical dark bands in palpi of adults [13]. Furthermore, these sibling species (A–D) were also confirmed through fixed inversions in the X-arm of polytene chromosomes viz. A = X+^a^,+^b^; species B = Xa,b; species C = Xa,+^b^; species D = X+a, b [13]. In Sri Lanka, the existence of *An. subpictus* sibling species A and B was first reported based on the single inversion (X+^a^/X^a^) on the X chromosome [14]. All four sibling species (A–D) have been reported in the country based on the previously reported morphometric identification characters [15]. Later, however, variations of morphological characters of *An. subpictus* sibling species were reported in various parts of the island [9,16]. Molecular analyses of internal transcribed spacer 2 (ITS2) and Cytochrome *c* Oxidase I (*COI*) have confirmed the presence of only two sibling species of the complex designating them provisionally as A and B [17]. Moreover, it has been recently reported that there are only two sibling species of *An. subpictus* in Thailand and more than one species in Indonesia [18]. This study has made use of crossing experiments, from which three distinct clades of *COI* phylogeny were determined as conspecific [18]. The work of Sindhania et al. [19] also suggests that there are three molecular forms of *An. subpictus* s.l. in the Indian subcontinent.

Speciation is a significant evolutionary phenomenon that occurs over time giving rise to new biological species [20,21]. The separation of members of different species based on genetic differences within sympatric populations occurs via reproductive barriers leading to subsequent reproductive isolation. The most probable reason for the appearance of sibling species or a species complex is the reconstruction of a genotype due to the reproductive isolation of two species that gives rise to mating incompatibility [5,22]. Sibling species are similar in morphology; however, they may accompany subtle morphological differences due to recently accumulated genetic mutations [23]. This state being in the early stages of reproductive isolation, sibling species may be deemed to have mutations in a least number and size of genes/intergenic regions that determines reproductive isolation leading to speciation [24]. These genomic islands of divergence, also known as speciation islands, are resistant to the homogenizing effects of gene flow [24]. For instance, as reported for *Anopheles gambiae* subtaxa M and S, which later elevated to species status as *Anopheles coluzzii* and *Anopheles gambiae* s. s. respectively, speciation islands showed a considerable gene flow [25]. However, it has later been shown that the entire 2L genomic island has undergone introgression during a period of rapid increase of voltage-gated sodium channel mutation (Vgsc-1014F), with apparently negligible impact on reproductive isolation [26]. In this respect, multigenic families in eukaryotic genomes may offer an appropriate degree of conservation for identification and inferring relationships among sibling species due to concerted evolution [27]. Ribosomal DNA (rDNA) ITS2 has proven to be an excellent molecular marker in uncovering sibling species, especially at lower taxonomic levels [28,29,30].

Located between the rRNA genes 5.8S, and 28S, ITS2 plays an important role in rRNA processing. The excision process of the ITS2 in the RNA transcript requires the formation of ITS2 secondary structures which seem to be conserved across most eukaryotes [31]. Further, a universal eukaryotic ITS2 secondary structure indicative of distinct hallmark features, was reported by Schultz et al. [32]. The hallmarks of a core ITS2 secondary structure consists of four helices with the helix III being the longest, a UGGU motif 5’ to the apex (deviations like UGGGU, UGG, or GGU have been described [33]) and a U-U mismatch in the helix II [32]. ITS2 secondary structures have been used for delimiting biological species based on the presence of compensatory base changes (CBCs) in the helix II or III of the structures [34,35,36]. CBCs occur in a paired region of a primary RNA transcript when both nucleotides of a paired site mutate while the pairing itself is maintained (e.g., G-C mutates to A-U) [37]. Hemi CBCs are transcripts in which only one nucleotide of a paired site mutates while the nucleotide bond is maintained (G-C mutates to A-C) [37]. Apparently, hemi CBCs can be regarded as intermediate products leading to CBCs. CBCs are associated with sexual incompatibility that taxa exhibiting at least one CBC in the conserved regions of the ITS2 secondary structures most likely belong to different biological species [36,38]. In fact, if there is one CBC, there is a ~0.93 probability that they belong to two different species [37]. However, since the CBC approach is a one-way tool, the absence of CBCs does not necessarily rule out that the two strains belong to one species but there is ~0.76 probability that they are the same species [37]. The occurrence of CBCs can be the underlying reason that ITS2 has appeared to be an effective indicator of species identification, accumulating mutations in reproductively isolated populations [39]. Thus, it has been shown that determining the presence of CBCs in ITS2 rRNA secondary structures is a reliable diagnostic tool of taxon analysis in prokaryotes placozoans [40] as well as higher eukaryotic lineages such as tick *Paramacrobiotus* [41], *Altica* beetles [42] and flowering plant *Corydalis* [43]. Furthermore, CBC analysis has been shown to play a supportive role in uncovering cryptic species in taxa including the marine protist *Amoebophrya ceratii* [44], marine diatom genus *Pseudo-nitzschia* [45] and skipper butterfly *Urbanus belli* [46]. Interestingly, CBC-based reproductive isolation at ITS2 has uncovered nine cryptic species within the mosquito *Anopheles longirostris* in Papua New Guinea [47].

In this context, it can be suggested that the controversy of the sibling species status of *An. subpictus* species complex in Sri Lanka may be resolved by predicting the reproductive isolation among its member species. Therefore, the objective of the present study was to analyze the intraspecies status of *An. subpictus* taxon in Sri Lanka based on ITS2 secondary structures and CBC analysis.

## 2. Materials and Methods

### 2.1. Mosquito Sampling 

**Figure 1:**
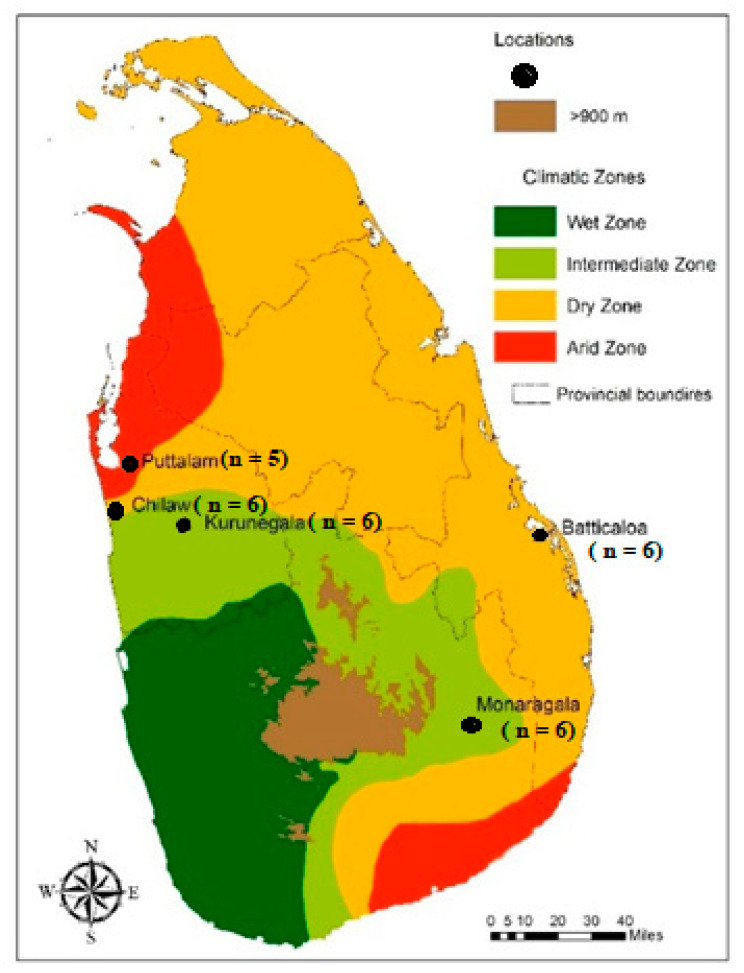
A map of Sri Lanka showing sampling sites: Puttalam, Chilaw, Kurunegala, Batticaloa and Monaragala [12].

Wild engorged female mosquitoes were collected (n = 128) during 2011–2014, with the assistance of entomological assistants, from five localities in Sri Lanka (Figure 1); Monaragala (6°87′27.02″ N, 81°35′06.70″ E), Kurunegala (7°48′71.00″ N, 80°36′49.95″ E), Puttalam (8°03′00.57″ N, 79°83′11.14″ E), Chilaw (7°57′65.41″ N, 79°79′56.57″ E) and Batticaloa (7°72′04.50″ N, 81°70′10.86″ E) using Cattle-Baited Hut Collection (CBHC) and Cattle-Baited Net Collection (CBNC) methods. The mosquitoes were identified as *An. subpictus* using the standard mosquito identification keys [48]. Under laboratory conditions, they were facilitated to lay eggs and the F_1_ progeny adults were obtained. However, the F_1_ adults were not identified for their sibling species status as described in Suguna et al. [13], due to the morphological polymorphism observed in all life cycle stages.

As shown in Figure 1, the location-wise distribution of the F_1_ mosquitoes used in the molecular analysis (n = 29) encompassed 6 mosquitoes from each of the locations in Monaragala, Kurunegala, Batticaloa, Chilaw and 5 mosquitoes from Puttalam.

### 2.2. DNA Extraction and PCR Amplifications 

Genomic DNA of F_1_ mosquitoes were extracted using a phenol-chloroform extraction method [49]. The ITS2 region was amplified using previously used PCR primers, reaction mixes and thermal profile [16] and sequenced at Macrogen Inc., Seoul, South Korea. The sequences were assembled using DNA Baser Sequence Assembler v3x (2014) and aligned using ClustalW multiple alignment (https://www.genome.jp/tools-bin/clustalw) [50]. *An. subpictus* ITS2 sequences of the present study were deposited in GenBank (KP165072-KP165079)

### 2.3. Determination of Genetic Divergence of ITS2

Genetic polymorphism of ITS2 region was determined in terms of gaps/missing data, GC content, number of haplotypes, haplotype diversity and nucleotide diversity using the software DnaSP v6.12.01 [51]. Additionally, the web server, Spectral Repeat Finder (SRF) [52] was utilized to find ITS2-specific dimers, trimers, tetramers. The program MEGA-X was used in calculating Kimura-2-parameter (K2P) for all ITS2 haplotypes [53].

### 2.4. ITS2 Sequence Phylogeny

Phylogenetic analysis was performed for the generated ITS2 sequences (KP165072-KP165079) and NCBI-retrieved ITS2 sequences. However, for ITS2, selection of GenBank sequences for phylogeny construction was limited depending on the availability of full ITS2 length. *Anopheles annularis* was chosen to be the outgroup due to its phylogenetic distance from *An. subpictus*. Best-suited DNA substitution model was determined as the general time reversible model (GTR), using the programme jModelTest 0.1.1 [54]. ITS2 phylogeny was constructed by Maximum Likelihood (ML) method using MEGA-X with 1000 bootstrap replicates [53]. The estimated bootstrap values were reported as ML bootstrap percentages.

### 2.5. ITS2 Sequence-Structure Analysis

The aligned full-length ITS2 sequences were annotated using Hidden Markov Models (HMMs) [55], implemented in the ITS2 Database (http://its2.bioapps.biozentrum.uni-wuerzburg.de/) to determine the proper ITS2 borders. ITS2 secondary structures (Default settings at 37 °C) were constructed using the program RNAStructure v5.6 [56]. They were viewed using the program 4SALE v1.5 [57,58] and the minimum free-energy structure that fulfilled the hallmarks of the typical eukaryotic ITS2 secondary structure [36] was selected as the template for custom homology modeling of the other sequences. The ITS2 secondary structures were obtained by homology modeling using the ITS2 Database and were viewed by the program PseudoViewer v 3.0 [59]. Global multiple sequence-structure alignments were automatically generated in 4SALE [57,58], and ITS2 sequences and their respective secondary structures were aligned using a 12 × 12 ITS2 sequence-structure specific scoring-matrix. This was used in the ProfDistS v0.9.9 [60] to obtain the ITS2 sequence-structure specific profile neighbor-joining (PNJ) tree.

### 2.6. Analysis of Compensatory Base Changes (CBCs)

The ITS2 sequence-structure data set was further analyzed for the presence of CBCs. CBCs were identified using the CBCAnalyzer option implemented in 4SALE v1.7 [57,58]. Furthermore, the ITS2 secondary structures were manually analyzed and confirmed for the CBCs and for hemi-CBCs. For comparison purposes, CBCs were also analyzed for the closely related species, *An. subpictus, Anopheles vagus* and *Anopheles sundaicus* using eligible GenBank retrieved sequences (*An. subpictus*: EF601868, EF601870; *An. vagus*: FJ457631, FJ654649; *An. sundaicus*: GQ284824, AY662258, AF369560, AF369562, AF469857, AY662445, GQ480826. The CBC table was transferred from the 4SALE results.

## 3. Results

### 3.1. Annotation of ITS2 Reveals Two Types of An. subpictus

The ITS2 sequence data of the present study did not contain any ITS2-specific sequence motifs. Table 1 summarizes the ITS2 data which are represented by the GenBank accession numbers KP165072-KP165079. KP165072 represents 15 specimens of the study. The accessions, KP165073-KP165079, represent 14 specimens that gave a nucleotide diversity of 0.00059. The sequences represented by KP165072 showed a high genetic distance value (K2P = 0.1) with respect to the ITS2 sequences represented by KP165073-KP165079 (Table 2).

### 3.2. ITS2 Secondary Structures Reveal Complimentary Base Changes

**Figure 2:**
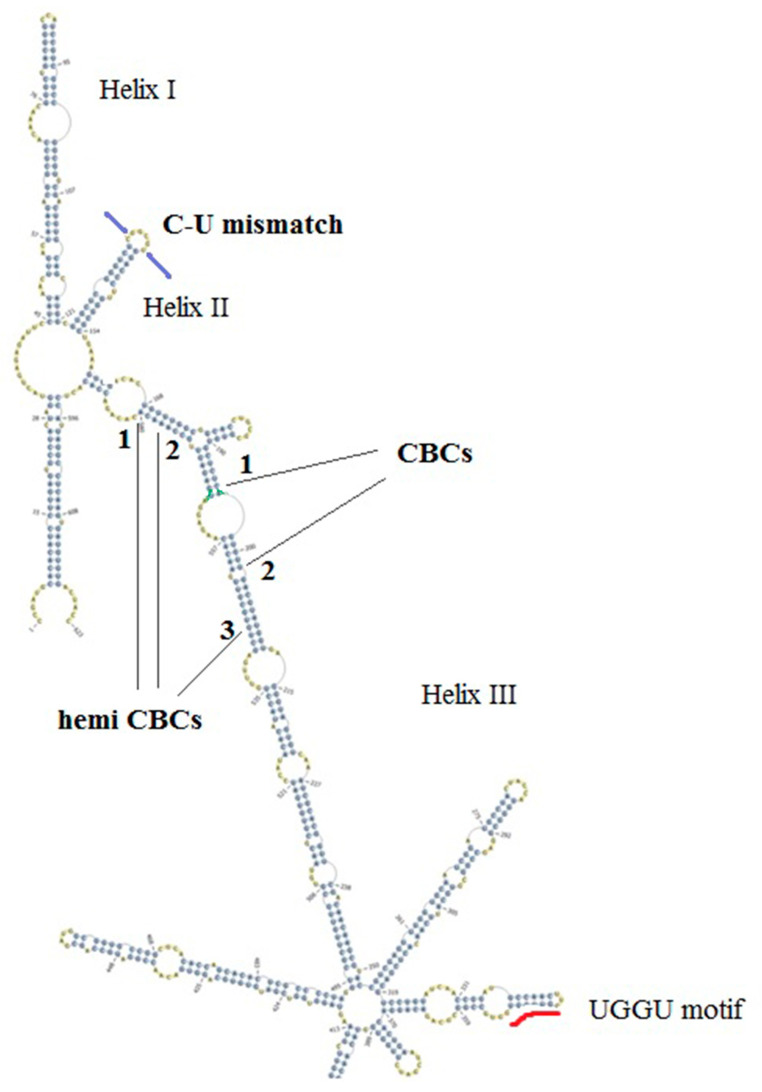
Three helix ITS2 secondary structure representing the 575 bp sized ITS2 sequences in the study. C-U mismatch is shown in helix II and a UGGU motif included in the distal part of helix III. Two compensatory base changes and the three hemi compensatory base changes are shown in numbers.

**Figure 3:**
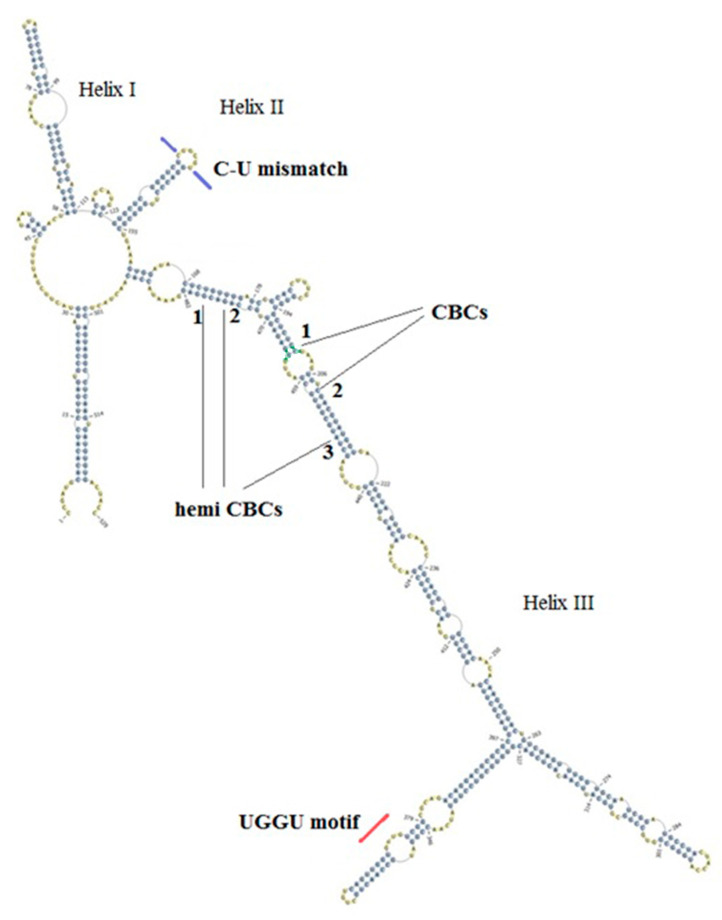
Three helix ITS2 secondary structure representing the 480 bp sized ITS2 sequences in the study. C-U mismatch is shown in helix II and a UGGU motif included in the distal part of the helix III. Two compensatory base changes and the three hemi compensatory base changes are shown in numbers.

Two representative ITS2 secondary structures were derived for *An. subpictus* specimens in the present study (Figure 2 and Figure 3). The structures were visibly different to each other in branching at the tip of helix III. The CBC analysis identified two CBCs (1. U-A to G-C; 2. A-U to G-C) and three hemi CBCs (1. G-U to A-U; 2. G-U to A-U; 3. A-U to G-U) in the helix III.

The number of CBCs detected against each ITS2 haplotype in the study are indicated in the upper diagonal of Table 2. For comparison purposes, CBC analysis among the closely related species is shown in Table 3. The presence of one or more CBCs between any two species imply that they are reproductively isolated, thus belonging to two species.

Despite the single haplotype corresponding to *An. subpictus* B (KP165072), the many haplotypes corresponding to *An. subpictus* A (KP165073-KP165079) showed slight differences in the secondary structures, in the branching pattern in helix III, however, CBCs were not detected within those structural changes.

### 3.3. ITS2 Sequence-Structure Analysis and ITS2 Sequence Phylogeny Reveal Two Distinct Clades for An. subpictus s.l.

**Figure 4:**
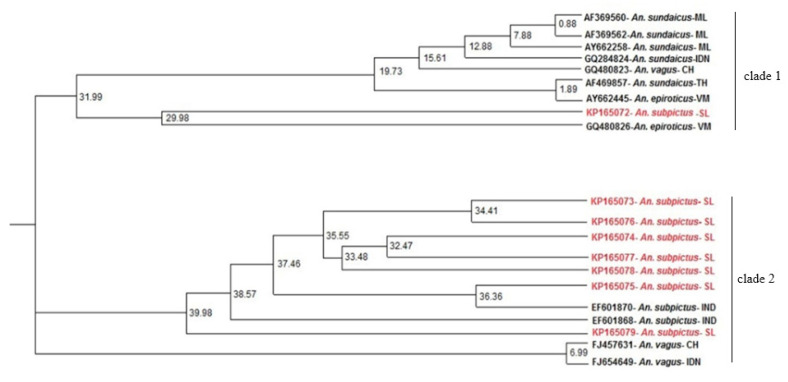
Profile neighbor joining tree for the study generated ITS2 sequence -structures constructed using ProfDistS 0.9.9. for the study-generated ITS2 sequences (KP165072-KP165079). Sequences retrieved from NCBI GenBank: AF369560, AF369562, AY662258—*Anopheles sundaicus* from Malaysia, GQ284824—*An. sundaicus* from Indonesia, GQ480823, FJ457631—*Anopheles vagus* from China, AF469857—*An. sundaicus* from Thailand, AY662445—*Anopheles epiroticus* from Vietnam, GQ480826—*An. sundaicus* from East Timor, EF601870, EF601868—*Anopheles subpictus* from India, FJ654649—*An. vagus* from Indonesia, and KC191825 and KC191826—*An. subpictus* A and B from Sri Lanka were included in the analysis.

Unlike neighbor joining which uses a distance-matrix for tree-building, profile-based neighbor joining relies on profiles in the form of sequence-structure alignments. Despite the minimal branch support, PNJ tree of the present study separated the *An. subpictus* sequences into two different clades (Figure 4).

**Figure 5:**
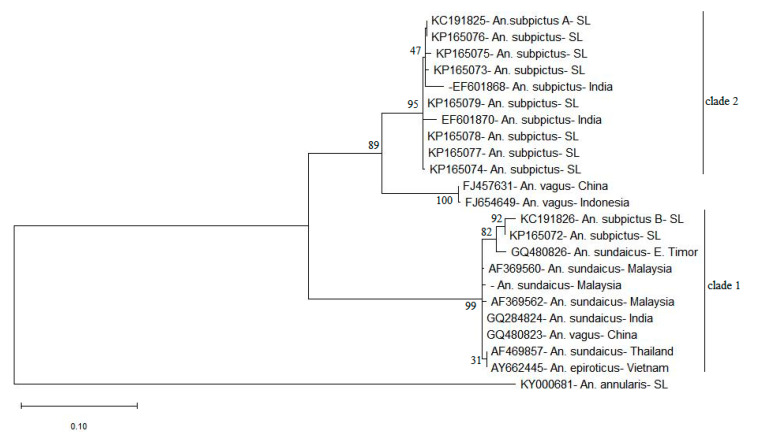
Maximum likelihood phylogenetic tree for study generated ITS2 sequences constructed using MEGA-X for 1000 bootstrap replicates. (Numbers at the nodes indicate bootstrap values). *Anopheles annularis* sequence (KY000681) served as the outgroup. Sequences retrieved from NCBI GenBank: AF369560, AF369562, AY662258—*Anopheles sundaicus* from Malaysia, GQ284824—*An. sundaicus* from Indonesia, GQ480823, FJ457631—*Anopheles vagus* from China, AF469857—*An. sundaicus* from Thailand, AY662445—*Anopheles epiroticus* from Vietnam, GQ480826—*An. sundaicus* from East Timor, EF601870, EF601868—*Anopheles subpictus* from India, FJ654649—*An. vagus* from Indonesia and KC191825, and KC191826—*An. subpictus* A and B from Sri Lanka were included in the analysis.

The ITS2 phylogenetic tree generated using ML method (Figure 5) also consisted of a similar tree topology to PNJ tree with the *An. subpictus* sequences dispersed into two different clades. In the ML tree, there is maximum support (bootstrap value 100) for the presence of these two clades. The sequence KP165072 clusters in the same clade 1 with the previously reported *An. subpictus* B in Sri Lanka (KC191826). Hence, it can be assumed that KP165072 (or clade 1 in the Figure 3) represents *An. subpictus* B in Sri Lanka. On the other hand, the sequences KP165073-KP165079 can be deemed to be ITS2 variants of *An. subpictus* A in Sri Lanka due to inclusion in the same clade 2 with the previously reported *An. subpictus* A (KC191825). Moreover, as shown by the clade 1 in phylogenetic trees (Figure 2 and Figure 3), *An. subpictus* B is related to *An. sundaicus* species. However, *An. subpictus* A is indicated as very distantly related to *An. subpictus* B.

## 4. Discussion

Being a secondary vector of malaria, *An. subpictus* is a potential key role player in possible future malarial episodes in Sri Lanka. In that regard, precise identification of sibling species in *An. subpictus* species complex plays a pivotal role in malaria elimination due to distinct variations in the biological characteristics such as vector competence [9]. However, sibling species status of *An. subpictus* species complex in the country remains controversial [14,15,17]. The present study provides a comprehensive molecular characterization of the ITS2 region of *An. subpictus* s.l. population in Sri Lanka. The ITS2 region was reliably recognized from the ITS2 Database as the 5.8S–28S proximal stem was conserved in all the sequences. Annotation of sequences by the HMM tool available in ITS2 Database indicated that the analyzed ITS2 regions were free of pseudogenes [61]. Determination of reliable borders for ITS2 through 5.8S and 28S flanking motifs confirmed two different ITS2 lengths for the specimens; 575 bp and 480 bp. However, the approximately 100 bp length difference revealed during ITS2 sequence annotation was not noticeable in PCR amplicons (data not shown) due to the reduced resolution of the 0.7% agarose gel used. The sequence conservation of form B, relative to A, with a nucleotide diversity of 0.00059 and a relatively high genetic distance (K2P = 0.1) clearly shows the genetic divergence of sequences that may have resulted due to different evolutionary constraints.

It has been reported in certain mosquito species, that longer ITS2 regions, as in the case with *An. subpictus* form A sequences in the present study, are due to occurrence of microsatellites, dimer, trimer, tetramer and pentamer repeats, polynucleotide microsatellites or typical CT repeats in 3’ end [62]. However, none was detected in any sequence of the current study. Conversely, sequences of longer ITS2 encompassed many deletions than the sequences with shorter ITS2, revealing a positive correlation between the occurrence of deletions and the spacer length.

Furthermore, the present study denies the previous finding that *An. subpictus* species B was present only in coastal localities of the island [14]. Additionally, found in the present study, record of both *An. subpictus* sibling species A and B from all the five locations of mosquito collection sites (data not shown) suggests the sympatric nature of the *An. subpictus* species complex in Sri Lanka. 

The ITS2 secondary structures derived in this study fulfilled the characteristic features of the universal eukaryotic ITS2 secondary structure [33]. However, they made only three helices as accordingly for mosquitoes [36] and the structures were congruent with the previously reported *Anopheles culicifacies* ITS2 secondary structures [63]. Of note is that the ITS2 secondary structures generated in the present study for both A and B sibling species were highly distinct from each other confirming the genetic distance suggested by the ITS2 sequence data. Altogether, both sequence and structure-based disparities indicate decreased introgression between the two sibling species, A and B.

However, this distantly related nature of *An. subpictus* A and B in Sri Lanka poses a question on their sibling species status whether they are siblings, despite the similar morphology. 

The presence of one or more CBCs in ITS2 secondary structures denotes sexual incompatibility and hence, it is an emerging tool for species delineation [38]. The presence of one CBC suggests that there is ~0.93 probability that there are two species [37]. Two CBCs and three hemi CBCs between the two types of ITS2 secondary structures in the present study suggest that the sibling species A and B are sexually incompatible. Although the 575 bp sized haplotypes (KP165073-KP165079), showed slight differences in the branching pattern in helix III (results not shown), the absence of CBCs suggest that there is approximately 0.76 probability that they belong to the same species [37] or sibling species.

The phylogenetic analyses of the present study are consistent with the previous studies conducted for ITS2 [15], *D3* [15] and *COI* [17], which clearly separated the species into two clades. Having had a sibling species status transiently updated from two to four within three decades, the present study confirms that the *An. subpictus* species complex in Sri Lanka is composed of two genetically distinct and sexually incompatible sibling species A and B. However, the genetic distance between the two (K2P = 0.1) can be considered ‘large’ at the sibling species level. In this context, it can be inferred that *An. subpictus* species complex consists of two sibling species A and B; however, with time, they are undergoing a significant evolutionary phenomenon which may lead to potential elevation as a distinct species. This idea is also consistent with our previous findings of intraspecific morphological variations at all life cycle stages [16], indicating an evolutionary process of morphological changes within sibling species. Additionally, considering the earlier characterizations of the complex, the four sibling species A-D [13], changing into two possible sibling species A-B [17], it is reasonable to assume an ongoing speciation process for the complex which may lead to evolution of a new species on the evolutionary time scale.

### Discrepancy on the Molecular Taxonomy of Anopheles subpictus, Anopheles sundaicus and Anopheles pseudosundaicus

The taxonomic status of *An. subpictus* has a complicated history. As reported in 1966, the then subspecies Indefinitus separated from Subpictus as a distinct species, namely *Anopheles indefinitus* [11]. The species *An. subpictus, An. sundaicus* and *An. vagus* belong to Pyretophorus Series. It has been shown that the species *An. vagus, An. indefinitus, An. subpictus* and *An. sundaicus* are hard to discriminate based on morphological characters [11]. Moreover, studies initiated after the Tsunami disaster in 2004 have revealed a new mosquito species, *Anopheles pseudosundaicus* in Southwestern India [64]. This species is also reported to have overlapped morphological characters with the *An. subpictus* s.l. and is taxonomically close to *An. subpictus* and *An. sundaicus* [64]. However, no molecular data have been published to date for *An. pseudosundaicus.*

*An. sundaicus* s.l. is an important vector of malaria in islands and coastal areas of Southeast Asia [65], but it has never been identified to occur in Sri Lanka so far. However, according to Surendran et al. [15], analysis of sequences of the ITS2 and D3 domain of rDNA have suggested that a majority that were identified morphologically as *An. subpictus* species B in the East coast of Sri Lanka were in fact the members of the Sundaicus complex based on genetic similarity to *An. sundaicus* s.l. [15]. The ITS2 phylogeny (ML) of the present study illustrated different clades for *An. subpictus* and *An. sundaicus* placed in close proximity (Figure 5). This was an evidence for the denial of the concept of genetic similarity of *An. subpictus* B and *An. sundaicus* but it indeed indicated close genetic affinity of *An. vagus* to *An. subpictus* A and *An. sundaicus* to *An. subpictus* B.

The CBC analysis indicated that there were no CBCs between Sri Lankan *An. subpictus* species B and *An. sundaicus* sequences analyzed (Table 3). Even though there is high chance (P = ~0.76) that they belong to the same species, theoretically [37], this may not apply here as *An. sundaicus* is morphologically different to *An. subpictus* due to presence of speckling, i.e., black and white scaled regions alternatively occurred in legs [65].

Additionally, ITS2 sequence comparison with sequences published by Wilai et al. [18] imply that *An. subpictus* s.l. in Thailand and Indonesia were distinct but genetically more related to *An. subpictus* sibling B in Sri Lanka [14] (Multiple sequence alignment shown in Figure S1). Considering this relativity of sibling B and the isolation of sibling A in the clade 2 of the ML tree (Figure 5), sibling A qualifies for potential separation as a distinct species. On the other hand, the CBC analysis revealed CBCs between *An. subpictus* sibling B and *An. subpictus* in India, suggesting that their reproductive isolation, hence, potential separation as a distinct species. However, further studies are required to confirm this speculation.

## 5. Conclusions

The sibling species composition of *An. subpictus* species complex in Sri Lanka has undergone significant updates since 1996. However, the present study confirms the early studies by Abhayawardana et al. [14] that *An. subpictus* s.l. in Sri Lanka is composed of two sibling species A and B. The presence of CBCs in their ITS2 secondary structures corroborate that they are two sexually incompatible species. The highly divergent ITS2 secondary structures also suggest that A and B are highly divergent from each other, thus they may be undergoing a speciation event to separate as a distinct species. However, this notion warrants further investigation in terms of analyzing more DNA loci including mitochondrial markers and single-copy DNA markers. Moreover, it is recommended to examine this mosquito complex for morphological, genomic and ecological variations and speciation information at different time points which may span from years to decades, to observe the outcome of the potential evolutionary processes ongoing with the species complex.

## Figures and Tables

**Table 1:** Overall summary of ITS2 parameters.

	ITS2	
Size	480 bp	575 bp
No. of sequences (n)	15	14
No. of segregating sites, (S)	0	1
No. of haplotypes (h)	1	2
Haplotype diversity (H_d_)	0	0.263
Nucleotide diversity (π)	0	0.00059

**Table 2:** Evolutionary divergence of sequences and the number of CBCs. Lower diagonal: Estimates of evolutionary divergence (K2P) for the study-generated ITS2 sequences; Upper diagonal: number of CBCs among study-generated ITS2 sequences.

	1	2	3	4	5	6	7
KP165072		2	2	2	2	2	2
KP165073	0.100		0	0	0	0	0
KP165074	0.100	0.004		0	0	0	0
KP165075	0.098	0.004	0.004		0	0	0
KP165076	0.098	0.003	0.004	0.002		0	0
KP165077	0.100	0.005	0.002	0.004	0.002		0
KP165078	0.098	0.005	0.002	0.004	0.002	0.000	
KP165079	0.098	0.002	0.002	0.002	0.000	0.000	0.000

**Table 3:** Matrix indicating compensatory base changes (CBCs) of closely related Anopheline species, *Anopheles subpictus, Anopheles vagus* and *Anopheles sundaicus.*

		*An. subpictus*		EF601868-*An. subpictus*-India	EF601870-*An.. subpictus* India	FJ457631-*An. vagus* China	FJ654649-*An. vagus*-Indonesia	AY662258-*An. sundaicus*-Malaysia	GQ480826-*An. sundaicus*-E. Timor	AF469857-*An. sundaicus*-Thailand	AY662445-*An. epiroticus*-Vietnam	GQ284824- *An. sundaicus- Indonesia*	GQ480823- *An. vagus*- China	AF369562- *An. sundaicus*- Malaysia	AF369560- *An. sundaicus*- Malaysia
		ITS2 575 bp	ITS2 480 bp												
*An. subpictus* *(Present study)*	ITS2 575 bp (A)	-	2	0	0	1	1	2	2	2	2	2	2	2	2
	ITS2 480 bp (B)	2	-	2	2	1	1	0	0	0	0	0	0	0	0
EF601868-*An. subpictus*-India		0	2	-	0	1	1	2	2	2	2	2	2	2	2
EF601870-*An. subpictus*-India		0	2	0	-	1	1	2	2	2	2	2	2	2	2
FJ457631-*An. vagus*- China		1	1	1	1	-	0	1	1	1	1	1	1	1	1
FJ654649-*An. vagus*-Indonesia		1	1	1	1	0	-	1	1	1	1	1	1	1	1
AY662258-*An. sundaicus*- Malaysia		2	0	2	2	1	1	-	0	0	0	0	0	0	0
GQ480826-*An. Sundaicus*-E. Timor		2	0	2	2	1	1	0	-	0	0	0	0	0	0
AF469857-*An. sundaicus*-Thailand		2	0	2	2	1	1	0	0	-	0	0	0	0	0
AY662445-*An. epiroticus*-Vietnam		2	0	2	2	1	1	0	0	0	-	0	0	0	0
GQ284824- *An. sundaicus- Indonesia*		2	0	2	2	1	1	0	0	0	0	-	0	0	0
GQ480823- *An. vagus*- China		2	0	2	2	1	1	0	0	0	0	0	-	0	0
AF369562- *An. sundaicus*- Malaysia		2	0	2	2	1	1	0	0	0	0	0	0	-	0
AF369560- *An. sundaicus*- Malaysia		2	0	2	2	1	1	0	0	0	0	0	0	0	-

-: no value.

## Supplementary Materials

The following are available online at https://www.mdpi.com/2075-1729/11/3/211/s1, Figure S1: Multiple sequence alignment of *An. subpictus* s.l. ITS2 sequences generated from the present study and the work by Wilai et al. [18].
