# Supplementary material for: Compensatory Base Changes Reveal Sexual Incompatibility among Members of the Anopheles subpictus Sensu Lato (Diptera: Culicidae) Species Complex in Sri Lanka
Source: Life (Basel). 2021 Mar 8;11(3):211. doi: 10.3390/life11030211 (PMC7998985; doi:10.3390/life11030211)

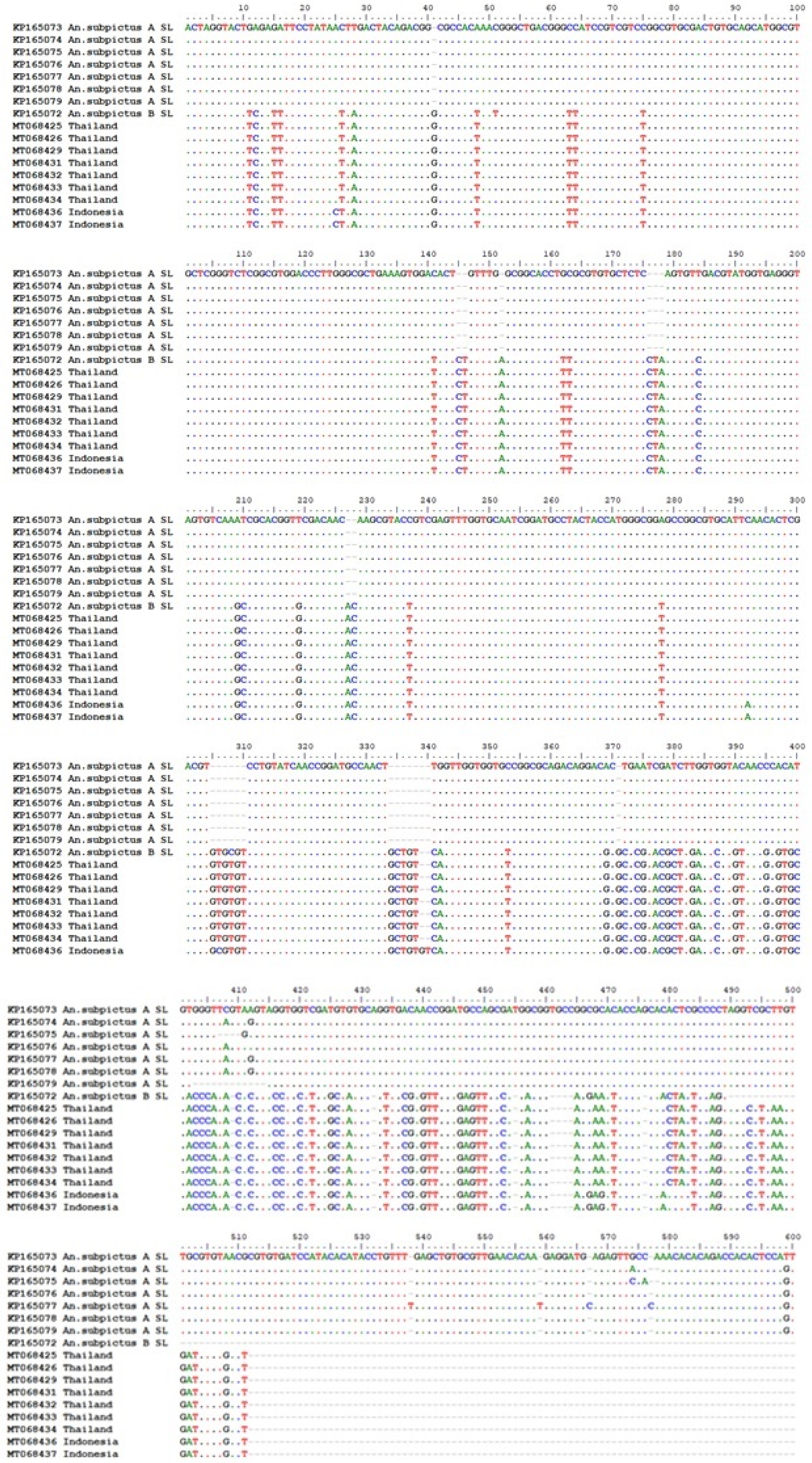

Supplement: Supplementary file 1 [file life-11-00211-s001.pdf]
